# Development of a new gastrointestinal endoscope forceps plug that can minimize gas leakage

**DOI:** 10.1002/deo2.268

**Published:** 2023-06-29

**Authors:** Tomo Ishida, Yoshinori Hayashi, Yohei Nose, Kohei Goto, Yuji Ishii, Takuro Saito, Kotarou Yamashita, Koji Tanaka, Kazuyoshi Yamamoto, Tomoki Makino, Tsuyoshi Takahashi, Yukinori Kurokawa, Hidetoshi Eguchi, Yuichiro Doki, Kiyokazu Nakajima

**Affiliations:** ^1^ Department of Next Generation Endoscopic Intervention (Project ENGINE) Osaka University Graduate School of Medicine Osaka Japan; ^2^ Department of Gastroenterological Surgery Osaka University Graduate School of Medicine Osaka Japan; ^3^ Department of Endoscope‐related Products, Sales Division TOP Corporation Tokyo Japan; ^4^ Medical and Welfare Development Division Castem Corporation Hiroshima Japan

**Keywords:** gas leak, gas leakage, gastrointestinal endoscope, plug, infection control

## Abstract

**Background and aims:**

In our previous study, we visualized and systematically evaluated gas leakage from the forceps plug of the gastrointestinal endoscope system using the Schlieren system. In order to minimize the potential risk of infection from gas leakage from the gastrointestinal endoscope, the development of a new forceps plug was considered urgent. In this study, we analyzed the structure of commercially available forceps plugs and attempted to develop new forceps plugs with improvements.

**Methods:**

Microfocus computed tomography was used to nondestructively analyze the structural changes that occur when forceps are inserted into a commercially available forceps plug. Based on the findings, the basic structure of the newly developed forceps plug was set. We examined the airtightness of these newly developed plugs using the Schlieren system and also compared their fractional resistance with those of commercially available plugs.

**Results:**

As a result of the nondestructive analysis, all of the commercially available plugs had a single valve, and the cleavage created in the valve during forceps, insertion was large in the case of plugs with slit‐type entrances. In the newly developed forceps plugs, all four types of plugs showed less gas leakage and similar or better usability compared to the commercially available plugs.

**Conclusions:**

The structural weaknesses of the existing gastrointestinal endoscopic forceps plugs were identified. Based on the findings, we froze the design of a prototype of a new forceps plug that was airtight and not inferior in usability compared to commercially available plugs.

## INTRODUCTION

With the global outbreak of severe acute respiratory syndrome coronavirus 2 (SARS‑CoV‑2), there has been increasing interest in assessing the risk of viral infection via leaking gas from medical devices.^1^, ^2^, ^3^ Since SARS‐CoV‐2 is thought to be mediated by particulate aerosols, which are finer than normal droplets, attempts have been made to visualize the behavior of the aerosols and assess the risk of virus exposure.^3^ We have previously reported that a certain amount of gas leakage from a gastrointestinal (GI) endoscopy system could be visualized by using Schlieren optical system and that the GI endoscopy system actually poses a risk of gas leakage.^4^ Also in the previous study, a certain amount of gas leakage was detected when forceps were inserted or removed into/from commercially available forceps plugs. On the other hand, there were differences in the amount of gas leakage depending on the types of plugs.

In this study, we first analyzed the structure of the commercially available forceps plugs non‐destructively to elucidate the mechanism of gas leakage. Furthermore, based on the obtained knowledge, we aimed to develop a new GI endoscope forceps plug that minimizes gas leakage and maintains usability.

## MATERIALS AND METHODS

### Experiment 1: a non‐destructive test of forceps plugs

#### Microfocus computed tomography

The structural changes of each plug during forceps insertion were examined in a non‐destructive manner using microfocus computed tomography (CT) imaging. Images of the structural changes of each plug during forceps insertion were acquired using an MCT225 micro‐CT scanner (Nikon Instech Co., Ltd., Tokyo, Japan) and R_mCT2 micro‐CT scanner (Rigaku Corporation, Tokyo, Japan). The imaging using the MCT225 micro‐CT scanner, commonly used for industrial applications (high‐resolution micro‐CT), was performed at an X‐ray setting of 90 kV, 90 μA, and 8.1 W with a 100 μm aluminum filter. The X‐ray source‐detector pair was fixed, with a 3 μm focal spot size, and the plugs and forceps fixed in the plastic container were placed on the rotating table at 1 cm from the X‐ray source. The subject was rotated through 360 degrees and received a 1.415 s exposure at each 0.129°increment. In addition, each angle was imaged with duplication in order to reduce scanning noise and the total exposure time was 132 minutes. The standard filtered back projection was used for reconstruction with square voxel sizes of 14 μm using VG Studio MAX (Volume Graphics GmbH, Heidelberg, Deutschland). The three‐dimensional image was constructed using the semi‐automated image processing software, IMARIS (Bitplane AG, Zurich, Switzerland).

#### Plugs and forceps

The differences in structural change among three types of commercially (CM) available plugs were investigated: CM‐1, universal type (711124; US Endoscopy, OH); CM‐2, reusable type (MB‐358; Olympus, Tokyo, Japan); and CM‐3, disposable type (MAJ‐1555, Olympus). Throughout the experiment, standard biopsy forceps (Radial jaw 4 2.0 mm; Boston Scientific, Marlborough, MA, US) were used.

### Experiment 2: device development

**FIGURE 1:**
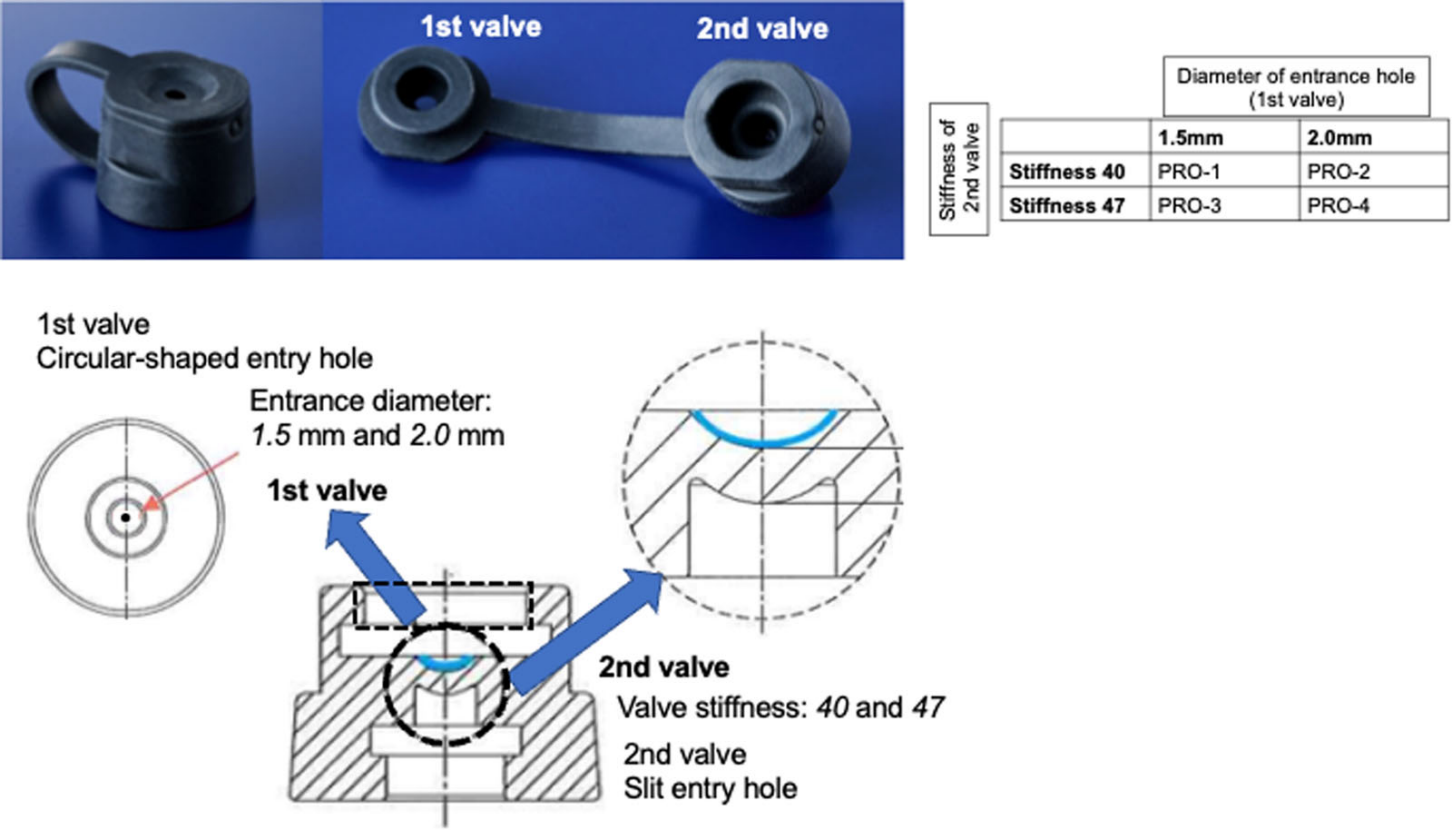
Newly developed prototype structure and lineup. Two different diameters of the first valve hole (1.5 or 2.0 mm) and two different stiffness of the second valve material (stiffness 40 and 47), 2 × 2, for a total of four different forceps plug prototypes were used.

The newly developed forceps plug was made to be a double‐valve structure. Figure 1 shows an overview of the double‐valve structure devised in this study. The forceps insertion hole of the first valve (the outer valve) was designed to be circular, similar to the universal type plug. The diameter of the hole of the first valve was set in two types, 1.5 and 2.0 mm. The second valve has a slit entry hole at the bottom of its mortar‐like structure. The stiffness of the second valve is also set in two types, 40 and 47° on a Type A durometer in accordance with JIS K 6253. Thus, four types of forceps plugs were made: Prototype (PRO)‐1 1.5 mm, 40°, PRO‐2) 2.0 mm, 40°, PRO‐3) 1.5 mm, 47°, and PRO‐4) 2.0 mm, 47° (thickness, stiffness) as shown in Figure 1.

### Gas leakage test

Procedures were performed on bench‐top simulators containing an explanted swine stomach.

#### The steady pressure automatically controlled endoscopy system

Steady pressure automatically controlled endoscopy system was constructed according to our previous reports but with several modifications.^5^, ^6^, ^7^ In order to ensure the reproducibility of the experiments, the intragastric pressure had to be kept constant at an arbitrary pressure, and each experiment was performed under the control of the steady pressure automatically controlled endoscopy system. Briefly, a detachable leakage‐proof device with an anti‐reflux valve and a Luer lock connection (Leak Cutter; Top, Tokyo, Japan) was connected to a standard endoscopic overtube (#16630; Top). A standard flexible GI endoscope (GIF‐Q260J; Olympus Medical Systems) was advanced into the stomach through the overtube. For CO_2_ insufflation, a dedicated CO_2_ insufflator (GW‐200; Fujifilm) was connected to the side channel of the Leak Cutter. The intragastric pressure was controlled at 8 mmHg via GW‐200.

#### Schlieren optical system

We used a large, sensitive Schlieren optical system (System Schlieren, SS100; Kato Koken, Tokyo, Japan) based on a 10 cm diameter parabolic telescope mirror to image the gas flows from the forceps plugs of endoscopy. ^8^ The Schlieren system is located in a 10 × 10 × 3.6 m laboratory. Room air‐conditioning is turned off while imaging to provide a quiescent ambient atmosphere. The average air temperature during testing is set at 22°C. High‐definition 1024 × 1024‐pixel, 200 frame/s video records of gas leakages were captured by a digital SLR camera (Nikon D90, Tokyo, Japan) with a 1/40,000 shutter speed.

#### The procedure of inserting and removing forceps

**FIGURE 2:**
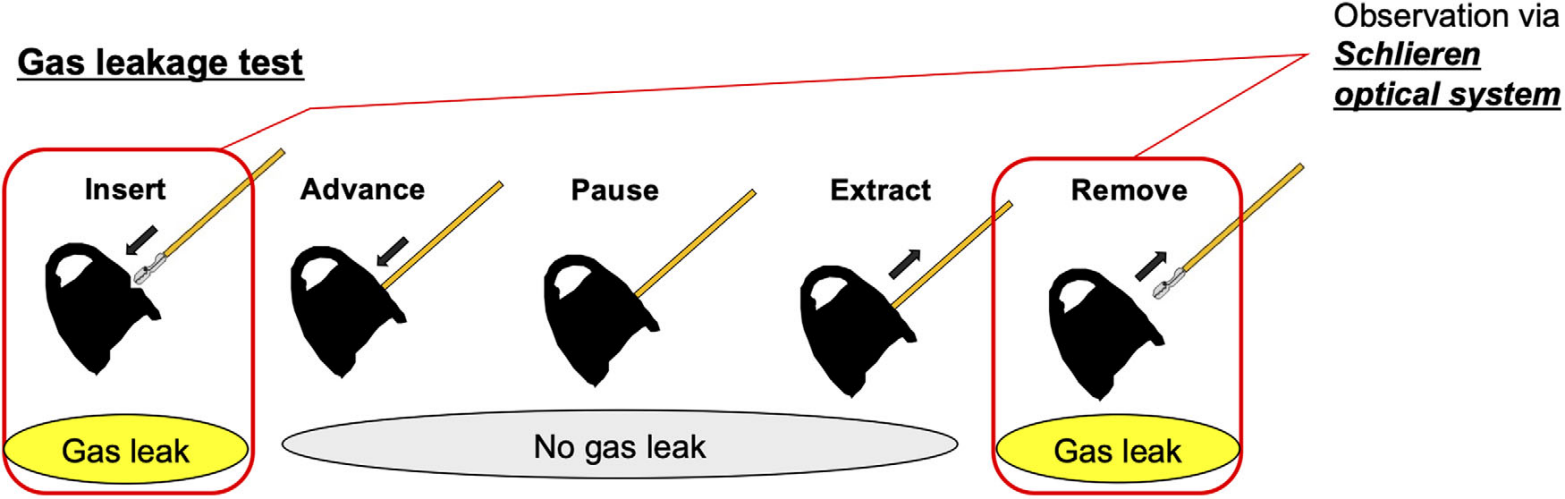
Device insertion and removal procedure in a gas leakage test. 1. Insert the device upright into the entrance hole. 2. Advance the device 1 cm/s keeping upright to the plug. 3. Advance 3 cm and pause for 1 s. 4. Extract the device 1 cm/s keeping upright to the plug. 5. Remove the device.

In order to minimize variability due to the procedure, the method of insertion and removal of the endoscopic instrument was standardized as follows. The forceps were inserted perpendicularly to the forceps plug and advanced vertically by 1 cm/s; after 3 cm insertion (i.e., after 3 s), the forceps were held in place for 1 s and then withdrawn vertically by 1 cm/s till complete removal. The same procedure was repeated five times for all plugs. The method of insertion and removal of the forceps is summarized in Figure 2.

### Friction test

**FIGURE 3:**
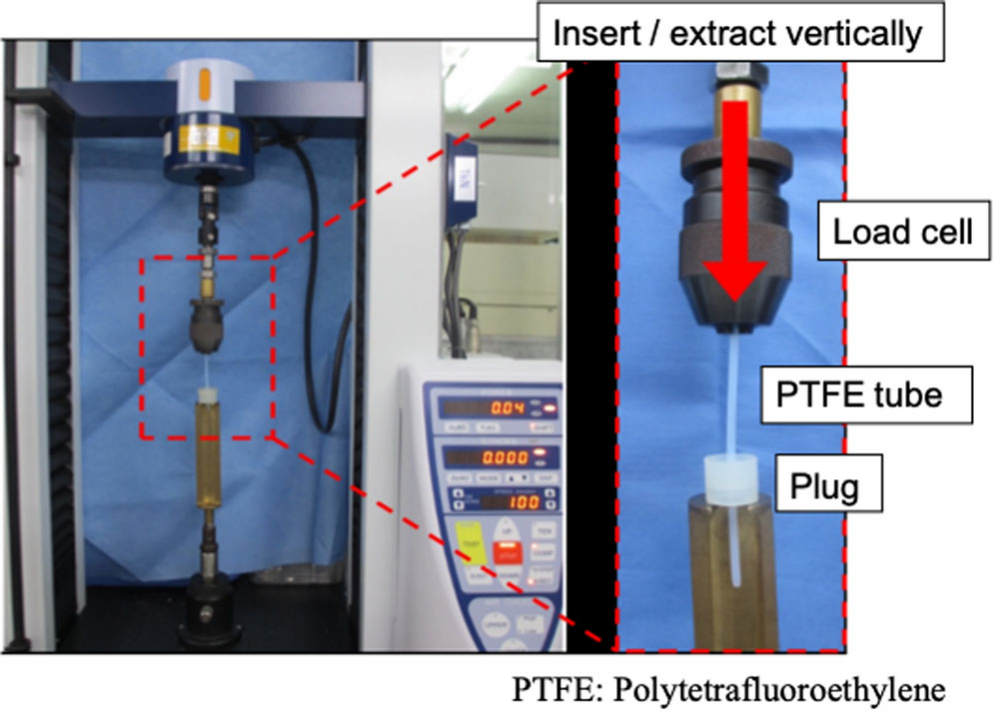
The method of friction test. A polytetrafluoroethylene (PTFE) tube (φ2.6 mm) was attached to the load cell of Strograf VGS (Toyoseiki, Co., Ltd, Tokyo, Japan). A forceps plug was attached at the position where the PTFE tube was inserted/removed straight on the movable axis of the load cell. The load cell was moved at a constant speed (100 mm/min in this case), and the friction of the forceps stopper when the device was inserted/removed was measured.

The insertion and extraction friction of each forceps plug was measured using Strograf VGS (Toyoseiki, Co., Ltd, Tokyo, Japan; Figure 3). First, a silicon tube (φ2.6 mm) was attached so that it is straight on the movable axis of the load cell. A forceps plug was attached at the position where the polytetrafluoroethylene tube was inserted/removed straight on the movable axis of the load cell. The load cell was moved at a constant speed (100 mm/min in this case), and the friction of the plug when the device was inserted/removed was measured. Since the moment of insertion/removal varied extremely widely for each trial, the measured value that became constant 1–2 s after the start of insertion/removal was recorded as the friction value. Ten tests were conducted for each plug, and the average value was compared to the CM‐2 plug, the most commonly used plug in Japan.

### Statistical analysis

Data were analyzed with JMP15 software (SAS Institute Inc., Cary, NC, US). The Mann–Whitney *U* test was used for group comparisons of continuous data.

### Ethical approval

This research is not medical research on human subjects and does not require IRB approval or written consent.

## RESULTS

### Experiment 1

**FIGURE 4:**
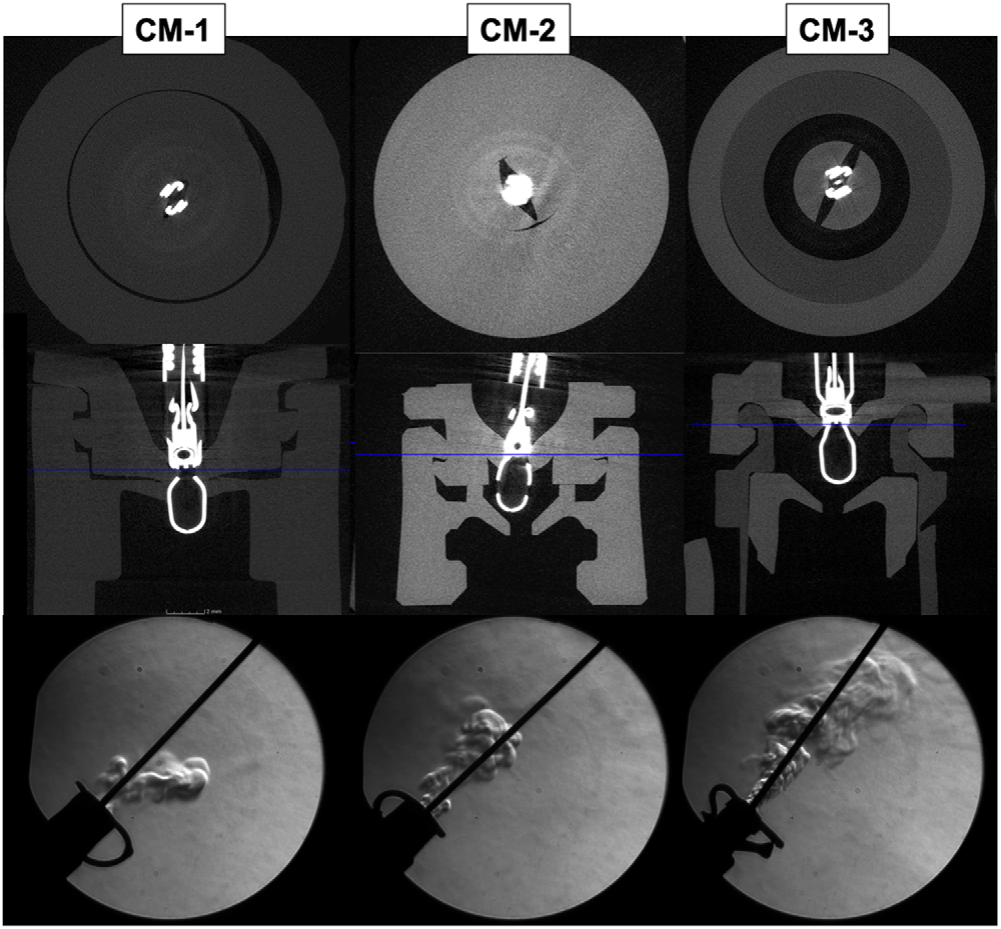
Non‐destructive examination for biopsy plugs using microfocus computed tomography and: Upper: axial view; Middle: coronal view; Lower: a typical image of gas leakage captured by the Schlieren system when forceps are inserted into each plug.

The structural changes of each forceps plug during forceps insertion were successfully demonstrated in a nondestructive fashion (Figure 4).　All of the commercially available forceps plugs were functionally single‐valve structures. CM‐1, which had the least gas leakage in the previous study, had a circular insertion hole in the valve, whereas the other two plugs had a slit‐shaped hole with a large cleavage created during forceps insertion.

### Experiment 2

#### Gas leakage test

**FIGURE 5:**
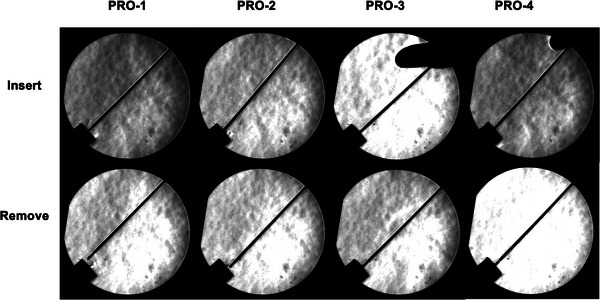
Typical Schlieren images of gas leakage during insertion/removal of forceps into/from each plug (above, during insertion; below, during removal).

Figure 5 shows representative images of the gas leakage observed with the Schlieren optical system when forceps are inserted into and removed from each plug. Unlike commercially available plugs (CM‐1, 2, and 3), no obvious quantifiable gas leakage was detected from either prototype (PRO‐1, 2, 3, and 4) during insertion or removal.

#### Friction test

**FIGURE 6:**
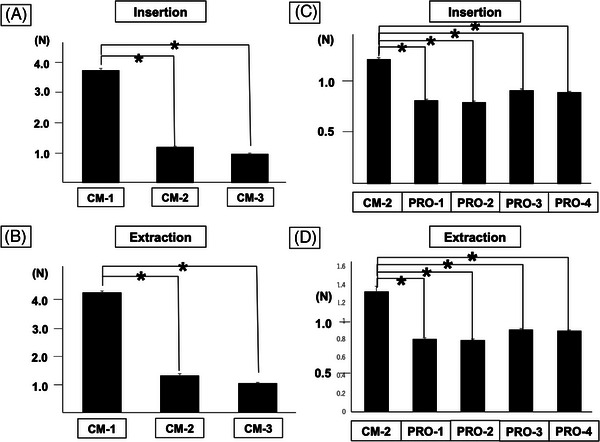
The results of the friction test. (a) Comparison of friction to forceps insertion in commercially available plugs. (b) Comparison of friction to forceps removal in commercially available plugs. (c) Comparison of forceps insertion friction between newly developed plug prototype and CM‐2. (d) Comparison of forceps removal friction between newly developed plug prototype and CM‐2. Data represent mean ± standard deviation (SD; *n* = 10), *p < 0.05.

Insertion friction (mean ± SD) for commercially available plugs (CM‐1, 2, and 3) was 3.77, 1.21, and 0.97 (N), respectively, with CM‐1 having significantly greater friction than the other two (*p* < 0.001 for both; Figure 6a). On the other hand, the extraction friction was 4.27, 1.33, and 1.06 (N), respectively, and was significantly higher in CM1 same as in insertion (*p* < 0.001 for both; Figure 6 b).　Insertion friction (mean ± SD) in the prototype plugs (PRO‐1, 2, 3, and 4) was 0.81, 0.80, 0.91, and 0.89 (N), respectively, all of which were significantly lower than that of CM‐2 (*p* < 0.001 for all; Figure 6c). On the other hand, the extraction friction was 0.80, 0.79, 0.91, and, 0.90, respectively, all significantly lower than that of CM‐2, as was the case with insertion (*p* < 0.001 for all; Figure 6d).

## DISCUSSION

The global outbreak of SARS‐CoV‐2 has prompted a reevaluation of the risk of viral infection in healthcare settings.^2^, ^3^ Since SARS‐CoV‐2 is believed to be transmitted via aerosol particles, which are smaller than droplets, attempts are being made to monitor the flow of aerosols in healthcare settings. In our previous study, we visualized the gas leakage from the forceps plug of a GI endoscope and found that a certain amount of gas leaked during the insertion and removal of forceps in the commercially available forceps plug. In another previous study from other groups, it was reported that the SARS‐CoV‐2 virus existed in gastric fluid and became a source of infection,^9^ therefore, the gas leakage from the forceps plug that we revealed was thought to be a threat that could not be overlooked in measures against viral infection. It has also been reported that the risk of viral infection cannot be eliminated even with complete personal protective equipment.^2^ Even under today's established standard precautions, gas leakage from forceps plugs must be addressed as an infection risk that should not be overlooked.

To investigate the mechanism of how gas leakage is caused, we non‐destructively analyzed the structural change when forceps were inserted into the commercially available plug in Experiment 1. We found two key points for minimizing gas leakage in this experiment. First, all commercially available forceps plugs are single‐valved, and gas leakage from the cleavage created during forceps insertion can leak directly to the outside world, directly leading to infection risk. It was suggested that the gas leakage to the outside world could be minimized if a double‐valve structure was used and the other valve could be closed when a cleavage was created in one valve. Another key point is the shape of the entrance of the valve. The plug that had relatively few gas leakage in the previous study (CM‐1) had a circular entrance hole, and the cleavage created by forceps insertion was smaller than that of plugs with slit‐type entrance holes (CM‐2,3). On the other hand, however, the small cleavage in the forceps plug with circular valves resulted in large friction suggesting that forceps insertion and handling was somewhat difficult. In view of the above, it was thought that it is important for the newly developed forceps plug to 1) have a double valve type, 2) have a circular entrance, and 3) minimize the friction during forceps manipulation.

The basic structure of the valve is shown in Figure 1, and the optimal valve thickness and material were determined in Experiment 2. As a result of the gas leakage test using the Schlieren optical device, it was found that all of the newly developed plugs clearly had less leakage (Figure 5 ).　In the previous study, gas leakage was seen only during the insertion and removal of the forceps, that is, when the head portion of the forceps passed through the valve, and no leakage was seen while the shaft of the forceps passed through the valve.^4^ The newly developed forceps plug has a double‐valve structure, so that even if a cleavage is created when the head is inserted into the first valve, the second valve is closed, so leakage will not be apparent. When the head reaches the second valve, the first valve has the forceps shaft through it, so leakage will not occur. It was thought that the structure was like an “airlock” in a spaceship.

On the other hand, the highly airtight structure with less gas leakage was expected to have strong friction during forceps manipulation, which would reduce usability. Actually, in commercially available plugs, the less leaky the plug, the higher the friction value tended to be (Figure 6a,b). However, friction tests showed that friction was significantly lower for both prototypes (PRO‐1, 2, 3, and 4) than for the commercially available plugs. Although the detailed structure and materials are confidential, we believe that we have succeeded in minimizing insertion friction by devising the internal structure. As a result, we succeeded in design‐freezing a prototype of a new forceps plug that is airtight and has non‐inferior usability compared to the commercially available forceps plug. Since no obvious leakage was evident in any of the prototypes, we intend to proceed with product development based on PRO‐2, which had the lowest insertion/extraction friction.

There are several limitations to this study. Firstly, all the experiments in this study were conducted in a benchtop environment using ex‐vivo models, and it is assumed that there are many differences from our daily clinical practice. In the future, it would be desirable to conduct validation on patients. Secondly, the evaluation of airtightness is qualitative, as it is not possible to quantify even the smallest leakage. However, on the contrary, the gas leakage in the new forceps plug is too trivial to be quantified, and it proves that it is not comparable to the commercial forceps plug in terms of air tightness.

In conclusion, we succeeded in designing a new GI endoscopic forceps plug with better usability, and minimized gas leakage compared to existing forceps plugs. We believe that this will greatly contribute to minimizing the risk of viral infection, including SARS‐CoV‐2.

We are now working on its regulatory clearance, commercialization, and actual use in our daily practice.

## CONFLICT OF INTEREST STATEMENT

None.

## Data Availability

The data that support the findings of this study are available on request from the corresponding author, Kiyokazu Nakajima. The data are not publicly available due to their containing information that could compromise the privacy of research participants.
